# Nivolumab as maintenance therapy following platinum‐based chemotherapy in *EGFR* ‐mutant lung cancer patients after tyrosine kinase inhibitor failure: A single‐arm, open‐label, phase 2 trial

**DOI:** 10.1111/1759-7714.15083

**Published:** 2023-09-12

**Authors:** Jiwon Kim, Chang‐Min Choi, Wonjun Ji, Jae Cheol Lee

**Affiliations:** ^1^ Department of Pulmonary and Critical Care Medicine, Asan Medical Center University of Ulsan College of Medicine Seoul South Korea; ^2^ Department of Oncology, Asan Medical Center University of Ulsan College of Medicine Seoul South Korea

**Keywords:** adverse events, immunotherapy, non‐small cell lung cancer, outcomes, tumor mutation burden

## Abstract

**Background:**

As the outcome of immunotherapy can be improved when concurrently or sequentially combined with cytotoxic chemotherapy or radiotherapy, we investigated the efficacy of immunotherapy maintenance following platinum‐based chemotherapy in epidermal growth factor receptor (*EGFR*)‐mutant non‐small cell lung cancer (NSCLC) after EGFR‐tyrosine kinase inhibitor (EGFR‐TKI) failure.

**Methods:**

In this prospective, open‐label, single arm phase 2 trial, we enrolled patients aged 18 years or older with *EGFR*‐mutant NSCLC, which progressed after first‐ or second‐line EGFR‐TKI. Patients received platinum‐based chemotherapy followed by nivolumab maintenance therapy. They were intravenously administered 240 mg of nivolumab every 2 weeks for 3 months followed by 480 mg every 4 weeks until disease progression or unacceptable toxic effects occurred. The primary endpoint was progression‐free survival (PFS). Secondary outcomes were overall survival (OS) and incidence of grade 3–4 treatment‐related adverse events (AEs).

**Results:**

We enrolled 26 patients between May 2020 and July 2021. The median PFS was 1.7 months (95% CI: 0.401–2.999 months). The median OS was 21.4 months (95% CI: 18.790–24.010 months) with 6‐ and 12‐month OS rates of 96.2% and 76.9%, respectively. The objective response rate was 7.7% (2/26) and disease control rate, 11.5% (3/26). The tumor mutational burden by next‐generation sequencing in blood was not related to the treatment outcomes. Grade 3–4 treatment‐related AEs occurred in four (15.4%) patients; the most frequent AE was increased alanine aminotransferase (7.7%).

**Conclusion:**

Nivolumab maintenance following platinum‐based chemotherapy did not show clinical benefits after EGFR‐TKI failure in patients with *EGFR*‐mutant NSCLC.

## Graphical abstract

Immunotherapy in patients with *EGFR*‐mutant lung cancer is not so effective. As the outcomes of immunotherapy when combined with chemotherapy can be improved, we investigated the efficacy of nivolumab therapy following platinum‐based chemotherapy in *EGFR*‐mutant NSCLC which progressed after EGFR‐TKI. In this phase 2 trial, it failed to show clinical benefit, with 1.7 months of median PFS.

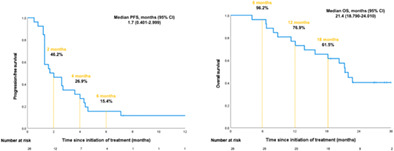

## INTRODUCTION

Lung cancer is the leading cause of cancer death worldwide,^1^, ^2^ but treatment outcomes have been improved with the development of targeted treatments for epidermal growth factor receptor (EGFR), anaplastic lymphoma kinase, and c‐ros oncogene 1. However, only ~30%–40% of patients with lung cancer have genetic mutations that can be addressed by targeted therapy,^3^ and disease progression is inevitable even for patients undergoing targeted therapies due to drug resistance.^4^, ^5^, ^6^ If there is disease progression after the use of targeted therapy, salvage treatment using cytotoxic chemotherapy or immunotherapy can be administered, but the prognosis is poor.^7^, ^8^

Several studies have shown that the response rate can be improved when immunotherapy is administered concurrently or sequentially with cytotoxic chemotherapy or radiotherapy.^9^, ^10^, ^11^ These can induce immunogenic cell death (ICD), which activates tumor‐specific immune responses potentiating the efficacy of immunotherapy.^12^ Moreover, the unfavorable tumor microenvironment (TME) could be altered by these therapies to recruit immune cells, making it more responsive to immunotherapy.^13^

**FIGURE 1:**
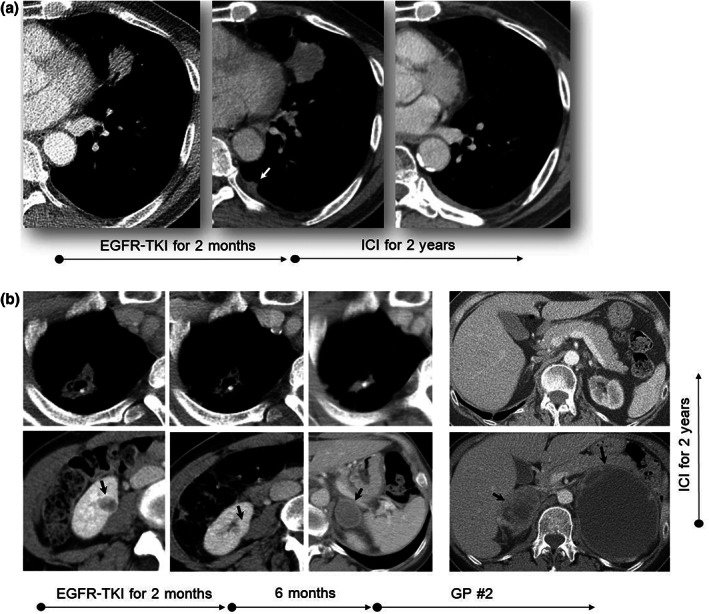
*EGFR*‐mutant cases with remarkable response to immunotherapy following platinum‐based chemotherapy after EGFR‐TKI failure. (a) Lung cancer with pleural metastasis (L858R+, PD‐L1 > 50%) showed primary resistance to EGFR‐TKI in a 70‐year‐old male patient. However, it responded very well to subsequent ICI, leading to CR after 2 years. (b) Lung cancer with kidney metastasis (L858+, PD‐L1 > 50%) improved with EGFR‐TKI administration in a 50‐year‐old female patient. However, the new left adrenal metastasis developed after 6 months. Subsequent gemcitabine + cisplatin was ineffective, with the increase of left adrenal metastasis and the new development of right adrenal metastasis. Unexpectedly, CR was achieved by following a therapy regimen of ICI for 2 years. CR, complete response; EGFR‐TKI, epidermal growth factor receptor tyrosine kinase inhibitor; GP, gemcitabine plus cisplatin; ICI, immune checkpoint inhibitor; PD‐L1, programmed death ligand 1.

Recently, we encountered some *EGFR*‐mutant cases that showed a remarkable response to immunotherapy following platinum‐based chemotherapy after EGFR‐tyrosine kinase inhibitor (TKI) failure, which initiated this study (Figure 1). Here, we investigated the efficacy of nivolumab maintenance following platinum‐based chemotherapy in patients with *EGFR*‐mutant non‐small cell lung cancer (NSCLC) with disease progression after EGFR‐TKI. We also explored the role of tumor mutation burden (TMB) in circulating tumor DNA on treatment outcomes.

## METHODS

### Study design and participants

This was a prospective, open‐label, single center, single‐arm, phase 2 trial performed at Asan Medical Center, Seoul, Republic of Korea. Eligible patients were aged at least 18 years with a pathologically proven diagnosis of *EGFR*‐mutant NSCLC that progressed after first‐ or second‐line EGFR‐TKI treatment. Patients were required to have an Eastern Cooperative Oncology Group performance state of 1 or lower, a measurable disease as per the Response Criteria in Solid Tumors (RECIST) criteria (version 1.1), and adequate major organ function. Patients were excluded if they had^1^ known small cell lung cancer transformation,^2^ multiple primary malignant tumors,^3^ residual adverse effects (AEs) of prior therapy or effects of surgery,^4^ current or past history of severe hypersensitivity to any other antibody products. The complete inclusion and exclusion criteria are provided in Table S1.

The study was conducted in accordance with the principles of the Declaration of Helsinki and the International Conference on Harmonization guidelines for Good Clinical Practice. The study protocol was approved by the institutional review boards of Asan Medical Center (IRB no.: 2019‐1493). All of the patients provided written informed consent.

### Procedures

The patients received platinum‐based chemotherapy followed by nivolumab maintenance therapy after EGFR‐TKI failure (Figure S1). Patients were administered 240 mg of nivolumab intravenously every 2 weeks for 3 months followed by 480 mg every 4 weeks until disease progression and/or unacceptable toxic effects occurred, or until 96 weeks (24 months) from the first dose of nivolumab.

Imaging assessments, including computed tomography or magnetic resonance imaging, of the brain were performed every 6 weeks (±7 days) for 6 months, and then every 12 weeks (±14 days) until disease progression. Tumor response and progression were evaluated as per protocol until disease progression according to RECIST version 1.1.

AEs were graded as per the National Cancer Institute Common Terminology Criteria for Adverse Events version 5.0 and monitored throughout the study for 30 days after the last dose of nivolumab or until a new treatment was started, whichever occurred first.

Blood samples were collected to assess the blood TMB before the first dosing of nivolumab and at the first tumor assessment. More than 10 mutations per mega‐base was defined as high TMB.

### Outcomes

The primary endpoint was progression‐free survival (PFS), which was defined as the time from the day nivolumab was initiated until objective tumor progression or death. Secondary endpoints were the objective response rate (ORR) and overall survival (OS), which were also calculated from the initiation of nivolumab. The safety profile was assessed during the study period after the administration of nivolumab. Exploratory objectives included the assessment of blood TMB, which could affect the treatment response of this therapy.

### Statistical analysis

Continuous variables are described in mean (standard deviation) and median (IQR) values. Categorical variables are described as frequencies using counts and percentages. We used the Kaplan–Meier method to estimate PFS and OS and corresponding 95% CIs. For PFS, patients whose disease condition did not progress were censored at the date of their final tumor evaluation. For OS, patients who were still alive were censored at data cutoff. The statistical analysis was performed using SPSS version 28.

## RESULTS

### Patient characteristics

We enrolled 26 patients between January 2020 and December 2021, of whom one withdrew their consent during the study. The patient demographics and characteristics are shown in Table 1. The median age was 60.5 years (IQR: 54.8–65.5 years). There were nine (34.6%) male patients, 17 (65.4%) patients had never smoked, and the histology of all the patients indicated adenocarcinoma. The most common metastatic site was extra‐thoracic lymph node (26.9%) followed by bone (23.1%). Programmed death ligand 1 (PD‐L1) expression was negative in six patients (23.1%), and two patients (7.7%) exhibited high TMB. There were 18 (69.2%) patients with 19 deletion mutations, while L858R was detected in 19.2%.

**TABLE 1:** Baseline patient demographics and disease characteristics.

Characteristics	All patients (*n* = 26)
Median age (range)	60.5 (54.8–65.5)
Sex, *n* (%)	
Male	9 (34.6)
Female	17 (65.4)
Smoking history, *n* (%)	
Ever‐smoker	9 (34.6)
Never‐smoker	17 (65.4)
ECOG PS, *n* (%)	
1	26 (100.0)
Tumor type, *n* (%)	
Adenocarcinoma	26 (100.0)
Primary *EGFR* mutation	
19del	18 (69.2)
L858R	5 (19.2)
Others	3 (11.5)
PD‐L1, *n* (%)	
<1%	6 (23.1)
1%–49%	10 (38.5)
50% <	1 (3.8)
TMB, *n* (%)	
<10 mt/Mb	24 (92.3)
10 mt/Mb<	2 (7.7)
Initial disease status, *n* (%)	
I	1 (3.8)
II	0 (0.0)
III	1 (3.8)
IV	24 (92.3)
Metastasis	
Single	7 (26.9)
Multiple	16 (61.5)
Metastasis site	
Extrathoracic lymph node	7 (26.9)
Bone	6 (23.1)
Brain	5 (19.2)
Liver	2 (7.7)
Adrenal gland	2 (7.7)
Others	11 (42.3)
Prior treatment	
Surgery	3 (11.5)
Radiotherapy	8 (30.8)
Chemotherapy	
First‐line EGFR‐TKI	
Afatinib	6 (23.1)
Geftinib	20 (76.9)
Second‐line EGFR‐TKI	
Osimertinib	12 (46.2)
Salvage chemotherapeutic agent before nivolumab maintenance	
Carboplatin + gemcitabine	19 (73.1)
Cisplatin + gemcitabine	7 (26.9)

Abbreviations: CCRT, concurrent chemoradiation therapy; ECOG PS, Eastern Cooperative Oncology Group performance state; EGFR, epidermal growth factor receptor; EGFR‐TKI, epidermal growth factor receptor tyrosine kinase inhibitor; PD‐L1, programmed death ligand 1; TMB, tumor mutation burden.

More than half (76.9%, 20/26) of the patients received gefitinib as the first‐line EGFR‐TKI treatment, and 12 (46.2%) received osimertinib as the second‐line treatment before platinum‐based chemotherapy.

### Efficacy

**FIGURE 2:**
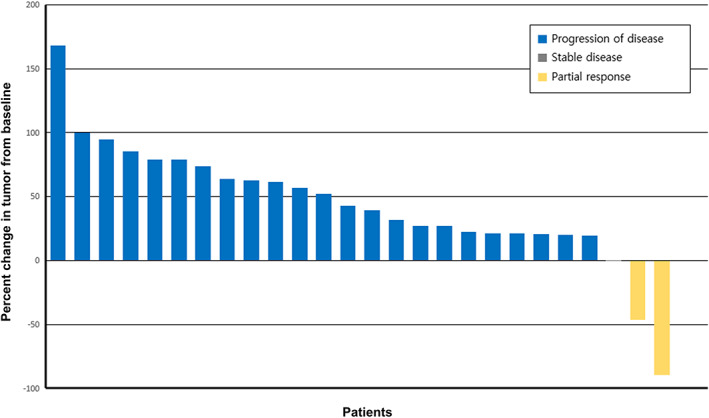
Waterfall plot of best change from baseline in 26 non‐small cell lung cancer patients.

**FIGURE 3:**
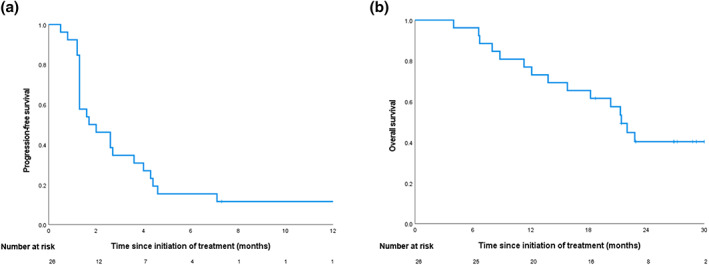
Progression‐free survival and overall survival of Kaplan–Meier curves of platinum‐based chemotherapy followed by nivolumab maintenance. (a) Progression‐free survival. (b) Overall survival.

The median follow‐up duration was 1.9 months (range, 1.3–4.3 months). According to the investigator assessment, partial response (PR) was achieved in two patients (7.7%), and stable disease (SD) was exhibited in one (3.8%), with no complete response (Table 2) (Figure 2). The median PFS was 1.7 months (95% CI: 0.401–2.999), and the median OS was 21.4 months (95% CI: 18.790–24.010) (Figure 3). The ORR and the disease control rate (DCR) were 7.7% and 11.5%, respectively. Based on the time of platinum‐based chemotherapy administration, the median PFS was 5.6 months (95% CI: 3.976–7.226), and the median OS was 24.8 months (95% CI: 22.651–26.949) (Figure S2). PD‐L1 expression and TMB were not related with the response and PFS (Table 3).

**TABLE 2:** Overall efficacy of nivolumab maintenance therapy following platinum‐based chemotherapy.

Response	All patients *n* = 26
RECIST responses, *n* (%)	
Complete response	0
Partial response	2 (7.7)
Stable disease	1 (3.8)
Progressive disease	23 (88.5)
Overall response, *n* %	2 (7.7)
Disease control, *n* %	3 (11.5)
PFS	
Median PFS, months (95% CI)	1.7 (0.401–2.999)
2‐month PFS, %	46.2
4‐month PFS, %	26.9
6‐month PFS, %	15.4
OS	
Median OS, months (95% CI)	21.4 (18.790–24.010)
6‐month OS, %	96.2
12‐month OS, %	76.9
18‐month OS, %	61.5

Abbreviations: CI, confidence interval; OS, overall survival; PFS, progression‐free survival; RECIST, response criteria in solid tumors.

**TABLE 3:** Comparison of baseline characteristics between patients in the responder and nonresponder groups in nivolumab maintenance therapy.

Characteristics	Responder (*n* = 3)	Nonresponder (*n* = 23)	*p*‐value
Median age (range)	58.0	61.0 (54.0–67.0)	0.736
Male, *n* (%)	0	9 (39.1)	0.529
Ever‐smoker, *n* (%)	0	9 (39.1)	0.529
Metastasis			>0.999
Single	0	7 (30.4)	
Multiple	1 (33.3)	15 (65.2)	
First‐line EGFR‐TKI			>0.999
Afatinib	0	6 (26.1)	
Geftinib	3 (100.0)	17 (73.9)	
Second‐line EGFR‐TKI			0.580
Osimertinib	2 (66.7)	10 (43.5)	
Salvage chemotherapeutic agent before nivolumab maintenance			0.167
Carboplatin + gemcitabine	1 (33.3)	18 (78.3)	
Cisplatin + gemcitabine	2 (66.7)	5 (21.7)	
Primary *EGFR* mutation			>0.999
19del	3 (100.0)	15 (65.2)	
L858R	0	5 (21.7)	
Others	0	3 (13.0)	
PD‐L1, *n* (%)			0.637
<1%	0	6 (26.1)	
1%–49%	1 (50.0)	9 (39.1)	
50%<	0	1 (4.3)	
TMB, *n* (%)			>0.999
<10 mt/Mb	3 (100.0)	21 (92.3)	
10 mt/Mb<	0	2 (8.7)	

Abbreviations: EGFR, epidermal growth factor receptor; PD‐L1, programmed death ligand 1; TMB, tumor mutation burden.

### Safety

AEs were reported by 13 (50.0%) patients. Treatment‐related AEs were mainly grade 1 or 2. Five (19.2%) patients had grade 3–4 AEs and two (7.7%), serious AEs (SAEs). The most common grade 3–4 AEs were laboratory abnormalities, especially increased alanine aminotransferase (7.7%) (Table 4). Nivolumab administration was adjusted or discontinued in seven (26.9%) patients because of AEs. The treatment‐related events leading to discontinuation reported in our study were polymyositis, ocular myasthenic crisis, and hyperglycemia.

**TABLE 4:** Treatment‐related adverse events of nivolumab maintenance therapy.

Adverse event	Any grade, *n* (%)	3–4 grade, *n* (%)	Serious AE, *n* (%)
Laboratory abnormalities			
Anemia	1 (3.8)	1 (3.8)	0
Glucose	1 (3.8)	1 (3.8)	1 (3.8)
Total cholesterol	1 (3.8)	0	0
Aspartate aminotransferase	5 (19.2)	1 (3.8)	0
Alanine aminotransferase	4 (15.4)	2 (7.7)	0
Alkaline phosphatase	2 (7.7)	0	0
γ‐glutamyl transferase	1 (3.8)	0	0
Lactate dehydrogenase	1 (3.8)	0	0
Other adverse events			
Nausea	3 (11.5)	0	0
Myalgia	2 (7.7)	0	0
Dyspnea	1 (3.8)	0	0
Rash	3 (11.5)	0	0
Enlarged lymph node	1 (3.8)	1 (3.8)	1 (3.8)
Thyroid dysfunction	2 (7.7)	0	0
Polymyositis	1 (3.8)	1 (3.8)	1 (3.8)
Ocular myasthenia gravis	1 (3.8)	1 (3.8)	1 (3.8)
Macular edema	1 (3.8)	0	0
Pneumonitis	0	0	0

## DISCUSSION

It is well‐known that immunotherapy in *EGFR*‐mutant lung cancer patients is less efficacious than in patients with *EGFR* wild‐type lung cancer.^14^, ^15^, ^16^, ^17^, ^18^ It has previously been reported that low PD‐L1 expression might be related to the poor response to immunotherapy in *EGFR*‐mutant lung cancer patients.^19^ However, the role of PD‐L1 does not appear to be clear because some studies found that the high PD‐L1 expression driving immune escape was more common in *EGFR*‐driven lung cancer.^20^, ^21^ TMB, another predictive marker of immunotherapy, was found to be lower in *EGFR*‐mutant lung cancer than in lung cancer with wild‐type *EGFR*.^22^ When considering that TMB is obviously associated with smoking status and that *EGFR*‐mutant lung cancer is mostly found in nonsmokers, it is probable that low TMB in *EGFR*‐mutant lung cancer could affect the efficacy of immunotherapy.

Despite the negative impact of *EGFR* mutation in immunotherapy, two cases shown in Figure 1, suggest that a subset of *EGFR*‐mutant lung cancers could respond well to immunotherapy. According to the study by Chen et al., the immune‐desert and immune‐excluded phenotypes of TME are unresponsive to immunotherapy.^13^ Because *EGFR*‐mutant lung cancer tends to have uninflamed TME,^23^ an improvement of immunotherapy efficacy would require a change to inflamed TME. As our cases received cytotoxic chemotherapy just before immunotherapy, the platinum‐based doublet chemotherapy would change the TME, causing this unexpected, remarkable response. ICD induced by cytotoxic chemotherapy could also contribute to the potentiation of the immunotherapy efficacy.

However, this phase 2 study failed to show any improvement in outcomes compared with previous studies on second‐line cytotoxic chemotherapy following EGFR‐TKI. In an open‐label phase 3 study called CheckMate‐057, patients with nonsquamous NSCLC that had progressed during or after platinum‐based doublet chemotherapy received nivolumab. The study reported a median PFS of 2.3 months and a median OS of 12.2 months.^14^ In our study, the median PFS was 0.6 months shorter than that reported in the previous study, whereas the median OS was 21.4 months. Wu et al. reported a median OS of 10.3 months with second‐line therapy in patients with *EGFR*‐mutant lung cancer, based on the timing of platinum‐based chemotherapy.^8^ In our study, the median PFS was 5.6 months. In a subgroup analysis of the NEJ002 study, which included 71 patients who received platinum‐based chemotherapy after gefitinib failure, the OS was 28.9 months.^24^ Therefore, we have to acknowledge that the addition of cytotoxic chemotherapy prior to immunotherapy cannot enhance the efficacy in *EGFR*‐mutant lung cancer.

Some studies have suggested that a high TMB may predict a good clinical response in the treatment with immune check point inhibitors.^25^, ^26^, ^27^, ^28^ In an open‐label phase 2 trial, CheckMate‐568, which involved patients with advanced or metastatic NSCLC receiving immunotherapy, it was observed that the patients with a TMB of 10 or above exhibited an increased ORR.^29^ Furthermore, in the CheckMate‐227 trial, which evaluated the efficacy of the combination therapy of nivolumab and ipilimumab, the study aimed to assess the PFS in patients with a TMB of 10 or above.^30^ Moreover, other trials suggest better response rates and PFS in tumors can be achieved with a greater PD‐L1 expression.^28^, ^31^ In a pooled analysis of international phase 3 trials, nivolumab was associated with a higher response rate than chemotherapy (47% vs. 28%) and with a longer PFS (9.7 months vs. 5.8 months) in patients with a high TMB.^25^ Hira et al. reported that there was no direct correlation between TMB and PD‐L1 expression, but both variables had similar predictive powers for a good response to immune checkpoint inhibitors.^28^ However, in this study, when responder groups were categorized according to PR or SD status, there was no significant difference in TMB and PD‐L1 expression between the responder and nonresponder groups. All three patients in the responder group had TMB <10 mt/Mb, while two patients with high TMB showed a poor response.

The safety profile of nivolumab in this study was consistent with that in previous studies.^14^, ^32^, ^33^ In a randomized, international, phase 3 CheckMate‐017 trial, 36% of the patients received nivolumab for at least 6 months.^32^ Of those patients, 58% experienced AEs and 7% experienced SAEs. Similarly, in another phase 3 trial, CheckMate‐057, 10% of the patients experienced grade 3 or 4 AEs, and 7% patients experienced SAEs among 30% of patients who had received nivolumab for more than 6 months.^14^ The most frequent grade 3 or 4 AEs were fatigue, myalgia, and loss of appetite, and the SAE that caused drug discontinuation was pneumonitis. In the present study, 11.5% of the patients received nivolumab treatment for at least 6 months, and 19.2% experienced grade 3 or 4 AEs. The rate of grade 3 or 4 AEs seemed to be higher here than in other studies. In this study, most grade 3 or 4 AEs were associated with laboratory abnormalities, such as alanine aminotransferase, aspartate aminotransferase, and hemoglobin. A high incidence of liver function test (LFT) abnormalities was observed, along with increased frequencies of nausea, myalgia, and rash. The elevated frequency of LFT abnormalities in this study may be associated with the occurrence of skin and gastrointestinal toxicities resulting from the administration of immune checkpoint inhibitors. It is noteworthy that the incidence of SAEs did not significantly increase compared to other studies. Furthermore, pneumonitis was not observed among the patients enrolled in this study.

There has been ongoing research focused on evaluating the efficacy of combining immunotherapy with chemotherapy in *EGFR*‐mutant NSCLC. Recent studies have provided important findings in this area. In the Impower150 trial, combination strategies involving antiangiogenic agents demonstrated promising outcomes. The addition of atezolizumab to bevacizumab and chemotherapy as a first‐line treatment for metastatic nonsquamous NSCLC resulted in a significant improvement in both PFS and OS. This survival benefit was consistently observed in the subgroup analysis focused on patients with *EGFR* mutations.^34^ However, the phase 3 KEYNOTE‐789 study reported contrasting results regarding immunotherapy. The addition of pembrolizumab to chemotherapy in patients with TKI‐resistant, *EGFR*‐mutated metastatic nonsquamous NSCLC resulted in a reduction in both PFS and OS compared to pemetrexed and chemotherapy alone. Due to the study not meeting its primary endpoint, it was discontinued.^35^ Therefore, the results of the KEYNOTE‐789 study and our study align, suggesting that the response to immunotherapy may be suboptimal in patients with an *EGFR*‐mutation. However, there may be variations among different immunotherapy drugs.

In conclusion, nivolumab maintenance following platinum‐based chemotherapy was well‐tolerated and showed no new unexpected adverse events. However, it failed to show clinical benefits after EGFR‐TKI failure in patients with *EGFR*‐mutant lung cancer. The factors related to the enhanced efficacy of immunotherapy in *EGFR*‐driven lung cancer should be further investigated as it is certain that some patients with *EGFR*‐mutant lung cancer exhibit a remarkable and durable response to immunotherapy.

## AUTHOR CONTRIBUTIONS

Conceptualization: Chang‐Min Choi, Wonjun Ji, Jae Cheol Lee. Data curation: Jiwon Kim, Chang‐Min Choi, Wonjun Ji, Jae Cheol Lee. Formal analysis: Jiwon Kim, Wonjun Ji. Funding acquisition: Jae Cheol Lee. Investigation: Chang‐Min Choi, Wonjun Ji, Jae Cheol Lee. Methodology: Wonjun Ji, Jae Cheol Lee. Validation: Chang‐Min Choi, Wonjun Ji, Jae Cheol Lee. Visualization: Jiwon Kim, Wonjun Ji. Writing ‐ original draft: Jiwon Kim, Wonjun Ji. Writing ‐ review & editing: Chang‐Min Choi, Wonjun Ji, Jae Cheol Lee.

## CONFLICT OF INTEREST STATEMENT

The authors have no conflicts of interest to declare. The funders had no role in the design of this study, data collection, data analyses, data interpretation, writing of the manuscript, or the decision to publish the results.

## Supporting information


**Figure S1.** Study schema.

**Figure S2.** Progression‐free survival and overall survival of Kaplan–Meier curves after platinum‐based chemotherapy. (A) Progression‐free survival. (B) Overall survival.

**Table S1.** Inclusion and exclusion criteria.
