# Supplementary figures and images for: Nivolumab as maintenance therapy following platinum‐based chemotherapy in EGFR ‐mutant lung cancer patients after tyrosine kinase inhibitor failure: A single‐arm, open‐label, phase 2 trial
Source: Thorac Cancer. 2023 Sep 12;14(31):3080–8. doi: 10.1111/1759-7714.15083 (PMC10626224; doi:10.1111/1759-7714.15083)

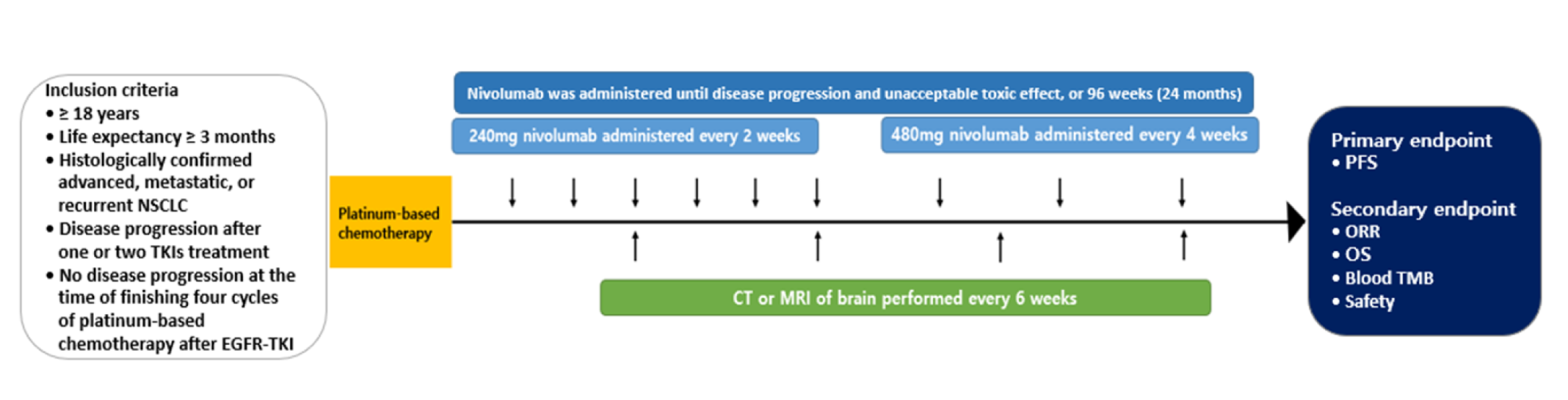

Supplement: Supplementary file 1 — Figure S1. Study schema. [file TCA-14-3080-s002.tiff]

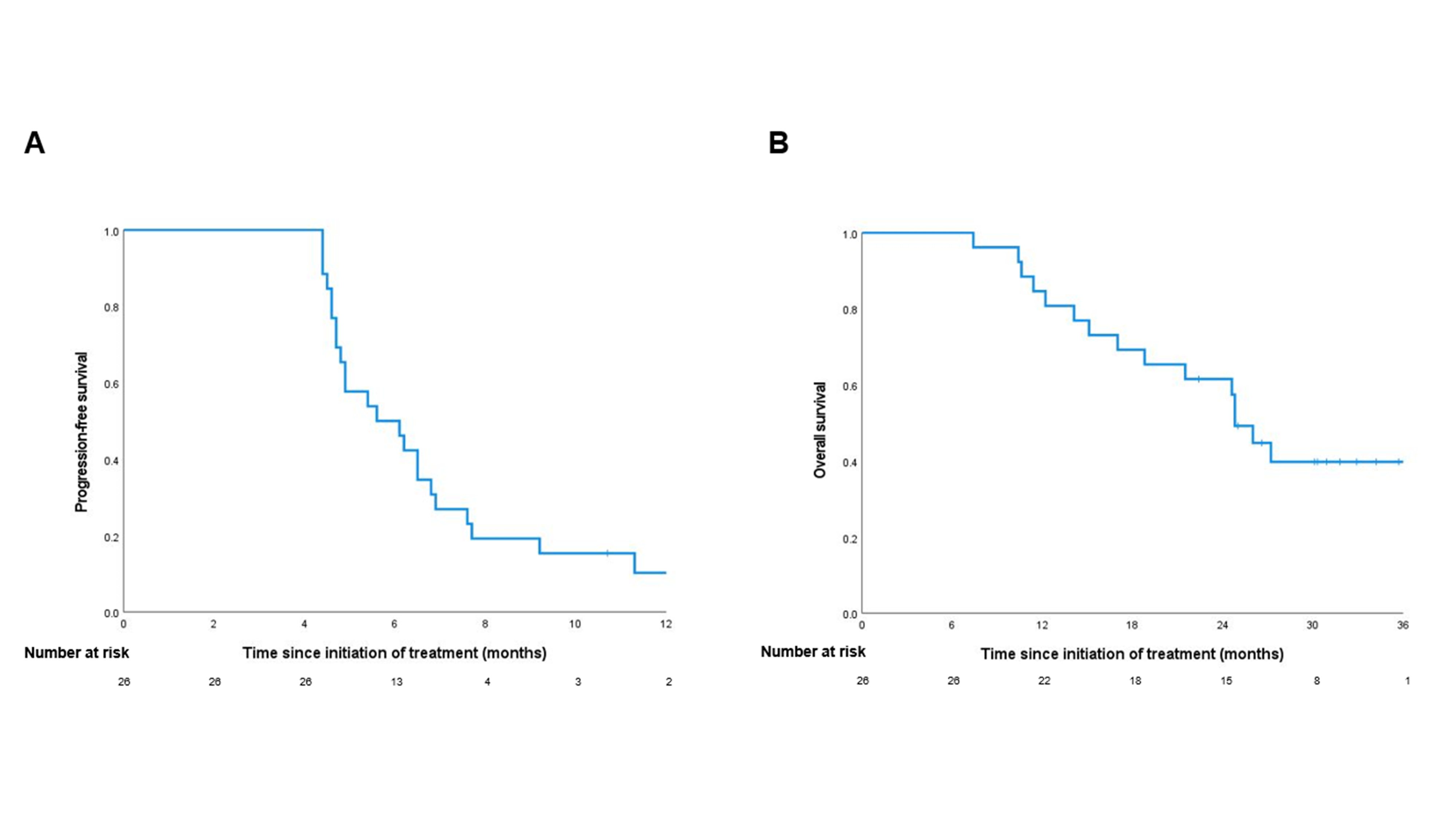

Supplement: Supplementary file 2 — Figure S2. Progression‐free survival and overall survival of Kaplan–Meier curves after platinum‐based chemotherapy. (A) Progression‐free survival. (B) Overall survival. [file TCA-14-3080-s001.tif]
