# Supplementary material for: Nivolumab as maintenance therapy following platinum‐based chemotherapy in EGFR ‐mutant lung cancer patients after tyrosine kinase inhibitor failure: A single‐arm, open‐label, phase 2 trial
Source: Thorac Cancer. 2023 Sep 12;14(31):3080–8. doi: 10.1111/1759-7714.15083 (PMC10626224; doi:10.1111/1759-7714.15083)
Supplement: Supplementary file 3 — Table S1. Inclusion and exclusion criteria. [file TCA-14-3080-s003.docx]

**Supplementary Table 1**. Inclusion and exclusion criteria

| **Inclusion criteria** |
| --- |
| - Age ≥18 y - ECOG performance status score 0 or 1 - Life expectancy ≥3 months - Histologically or cytologically confirmed advanced, metastatic, or recurrent NSCLC - Have at least one measurable lesion per the RECIST guideline version 1.1. - Disease had progressed after one or two TKIs treatment - Did not shown disease progression at the time of finishing four cycles of platinum-based chemotherapy after TKI therapy failure - Latest laboratory data met the following criteria. Of note, laboratory data will not be valid if the patient has received a granulocyte colony-stimulating factor (G-CSF) or blood transfusion within 14 days before testing. - WBC ≥2 x 103/μL and ANC ≥1,500/μL - Platelets ≥100 x 103/μL - Hemoglobin ≥9.0 g/dL - AST and ALT ≤3 fold the upper limit of normal (ULN) of the study site (or ≤5.0-fold the ULN of the study site in patients with liver metastases) - Total bilirubin ≤1.5-fold the ULN of the study site - Creatinine ≤1.5 fold the ULN of the study site or creatinine clearance >45 mL/min - Women of childbearing potential (including women with chemical menopause or no menstruation for other medical reasons) must agree to use contraception from the time of informed consent until 5 months or more after the last dose of the investigational product. Women must also agree not to breastfeed from the time of informed consent until 5 months or more after the last dose of the investigational product - Men must agree to use contraception from the start of the study treatment until 7 months or more after the last dose of the investigational product |
| **Exclusion criteria** |
| - SCLC transformation - Multiple primary cancers (with the exception of completely resected basal cell carcinoma, stage I squamous cell carcinoma, carcinoma in situ, intramucosal carcinoma, or superficial bladder cancer, or any other cancer that has not recurred for at least 5 years) - Patients with residual adverse effects of prior therapy or effects of surgery that would affect the safety evaluation - Current or past history of severe hypersensitivity to any other antibody products - Concurrent autoimmune disease or history of chronic or recurrent autoimmune disease. Patients with controlled asthma in the opinion of the investigator may be enrolled - Current or past history of interstitial lung. Patients with radiation pneumonitis may be enrolled if the radiation pneumonitis has been confirmed as stable (beyond acute phase) - Concurrent diverticulitis or symptomatic gastrointestinal ulcerative disease - Any metastasis in the brain or meninx that is symptomatic or requires treatment - Pericardial fluid, pleural effusion, or ascites requiring treatment - Uncontrollable, tumor-related pain - Transient ischemic attack, cerebrovascular accident, thrombosis, or thromboembolism (pulmonary arterial embolism or deep vein thrombosis) within 180 days before enrollment - History of uncontrollable or significant cardiovascular disease meeting any of the following criteria: - Myocardial infarction within 180 days before enrollment - Uncontrollable angina pectoris within 180 days before enrollment - New York Heart Association (NYHA) Class III or IV congestive heart failure - Uncontrollable hypertension despite appropriate treatment - Arrhythmia requiring treatment - Anticoagulant therapy for a disease - Uncontrollable diabetes mellitus - Systemic infections requiring treatment - Received systemic corticosteroids or immunosuppressants within 28 days before enrollment - Received chemotherapy within 14 days before enrollment - Undergone surgical adhesion of the pleura or pericardium within 28 days before enrollment - Undergone surgery under general anesthesia within 28 days before enrollment - Undergone surgery involving local or topical anesthesia within 14 days before enrollment - Received radiotherapy within 14 days before enrollment, or radiotherapy to bone metastases within 14 days before enrollment - Undergone CyberKnife or Gamma knife treatment 14 days at most before enrollment - Received any radiopharmaceuticals within 56 days before enrollment - History of a positive test result for any of the following: HIV Ag/Ab, HCV antibody, or HBs antigen with a detectable level of HBV-DNA - Women who are pregnant, breastfeeding, or possibly pregnant - Received any other unapproved drug (e.g., investigational use of drugs, unapproved combined formulations, or unapproved dosage forms) within 28 days before enrollment - Previously received nivolumab, anti-PD-1 antibody, anti-PD-L1 antibody, anti-PD-L2 antibody, anti-CD137 antibody, anti-CTLA-4 antibody, or other therapeutic antibodies or pharmacotherapies for the regulation of T-cells - Patients judged to be incapable of providing consent for reasons such as concurrent dementia - Current or past history of hypersensitivity to nivolumab |
